# Dynamic spatiotemporal analysis of indigenous dengue fever at street-level in Guangzhou city, China

**DOI:** 10.1371/journal.pntd.0006318

**Published:** 2018-03-21

**Authors:** Kangkang Liu, Yanshan Zhu, Yao Xia, Yingtao Zhang, Xiaodong Huang, Jiawei Huang, Enqiong Nie, Qinlong Jing, Guoling Wang, Zhicong Yang, Wenbiao Hu, Jiahai Lu

**Affiliations:** 1 School of Public Health, Sun Yat-Sen University, Guangzhou, Guangdong, China; 2 School of Public Health and Social Work, Institute of Health and Biomedical Innovation, Queensland University of Technology, Brisbane, Queensland, Australia; 3 Guangzhou Center for Diseases Control and Prevention, Guangzhou, Guangdong, China; 4 Department of Integrated Control and Prevention Management, Haizhu District Center for Diseases Control and Prevention, Guangzhou, Guangdong, China; 5 One Health Research Centre (School of Public Health), Sun Yat-Sen University, Guangzhou, Guangdong, China; 6 Key Laboratory of Tropical Disease Control (Sun Yat-Sen University), Ministry of Education, Guangzhou, Guangdong, China; 7 Key Surveillance Laboratory of Vector-borne Infectious Diseases, Haikou, Hainan, China; Beijing Institute of Microbiology and Epidemiology, CHINA

## Abstract

**Background:**

This study aimed to investigate the spatiotemporal clustering and socio-environmental factors associated with dengue fever (DF) incidence rates at street level in Guangzhou city, China.

**Methods:**

Spatiotemporal scan technique was applied to identify the high risk region of DF. Multiple regression model was used to identify the socio-environmental factors associated with DF infection. A Poisson regression model was employed to examine the spatiotemporal patterns in the spread of DF.

**Results:**

Spatial clusters of DF were primarily concentrated at the southwest part of Guangzhou city. Age group (65+ years) (Odd Ratio (OR) = 1.49, 95% Confidence Interval (CI) = 1.13 to 2.03), floating population (OR = 1.09, 95% CI = 1.05 to 1.15), low-education (OR = 1.08, 95% CI = 1.01 to 1.16) and non-agriculture (OR = 1.07, 95% CI = 1.03 to 1.11) were associated with DF transmission. Poisson regression results indicated that changes in DF incidence rates were significantly associated with longitude (β = -5.08, *P*<0.01) and latitude (β = -1.99, *P*<0.01).

**Conclusions:**

The study demonstrated that social-environmental factors may play an important role in DF transmission in Guangzhou. As geographic range of notified DF has significantly expanded over recent years, an early warning systems based on spatiotemporal model with socio-environmental is urgently needed to improve the effectiveness and efficiency of dengue control and prevention.

## Author summary

Dengue fever (DF) as a mosquito-borne viral disease remains a challenge for the prevention and control caused by the increased population, global development, human movement, and urbanization in the last five decades. The largest DF outbreak occurred with more than 40,000 cases in Guangdong in 2014 since DF re-emerged in China. The accurately spatiotemporal identification of DF transmission and the related socio-environmental factors are considered to be important for the strategy decision-making of the official government. This study first identified the spatiotemporal pattern and socio-environmental factors associated with DF occurrence at street and daily level in Guangzhou, China from 2006 to 2014, using spatiotemporal scan statistical methods. The results suggested that DF control should be targeted in the southwest of Guangzhou during autumn, particularly 75 high risk streets. We found that the aged population, floating population, low-education population and the non-agricultural population significantly contributed to the DF clustering risk at street level. Finally, a spread trend of DF toward southwest part of Guangzhou was noticed. These results could be implemented towards prevention and control measures of DF in high-risk areas in Guangzhou.

## Introduction

Dengue fever (DF) is a widespread vector-borne viral infectious disease which has a rapidly increase in infections, geographic distribution, and the severity cases[1]. The rapidly expanding global footprint of DF has evolved to a major public health problem due to increased geographical extension, climate changes, population growth and global travel in the last 50 years [2]. DF is endemic and has been reported in more than 100 countries including the southeast Asia, the Americas, the western Pacific, Africa [3]. 3.9 billion people are at the potential risk of DF in these endemic regions [4]. The high economic burden brought could not been neglected [5].

Historically, DF has re-emerged in China in 1978, from its first appearance in Foshan city of Guangdong province and then subsequently it has been reported in other areas such as Guangdong, Guangxi province and Hainan island after 32 years [6]. Since then, DF outbreak and epidemics were reported every year affecting several thousands of people, predominantly in the southeast coastal regions including Hainan, Guangxi, Fujian, Zhejiang and Yunnan provinces [7]. It was assumed that the large-scale epidemics occurred before 1990s was due to the imported dengue virus [8]. During the period 1978–2008, a total of 655,324 cases including 610 deaths were recorded by Guangdong province Health Department. Vector-borne scientists have predicted that DF could potentially become an endemic disease in China [9]. For example, in 2014, a large outbreak with more than 37,000 cases has occurred in Guangzhou city [10].

Due to the lack of effective vaccine and antiviral treatment, vector control is considered as a useful measure towards prevention of dengue disease [11]. DF epidemics in the different districts appeared not homogenous, due to the change of the transmission pattern of spatial and time [11]. However, the spatial clusters, socio-environmental factors at the new and smallest administrative unit (street level) and the temporal cluster at daily level in Guangzhou have not been explored in this epidemic regions. To help decision-makers or policy-makers in targeting the prevention and control areas and reduce the economic burden, vector control techniques could be selectively applied at high-risk areas or clusters of DF. Hence, this study aimed to examine the spatiotemporal pattern of DF using spatiotemporal scan technique at street-level[10,12,13], to identify the socio-environmental risk factors of DF and to explore the spread of DF over the study period for improving prevention and control of DF and guiding to future study.

## Methods

### Ethics statement

Ethical approval for this project was approved by Sun Yat-Sen University Ethical Review Committee (Approval No: 2015024) and all of the data analyzed were anonymized.

### Study setting

**Fig 1:**
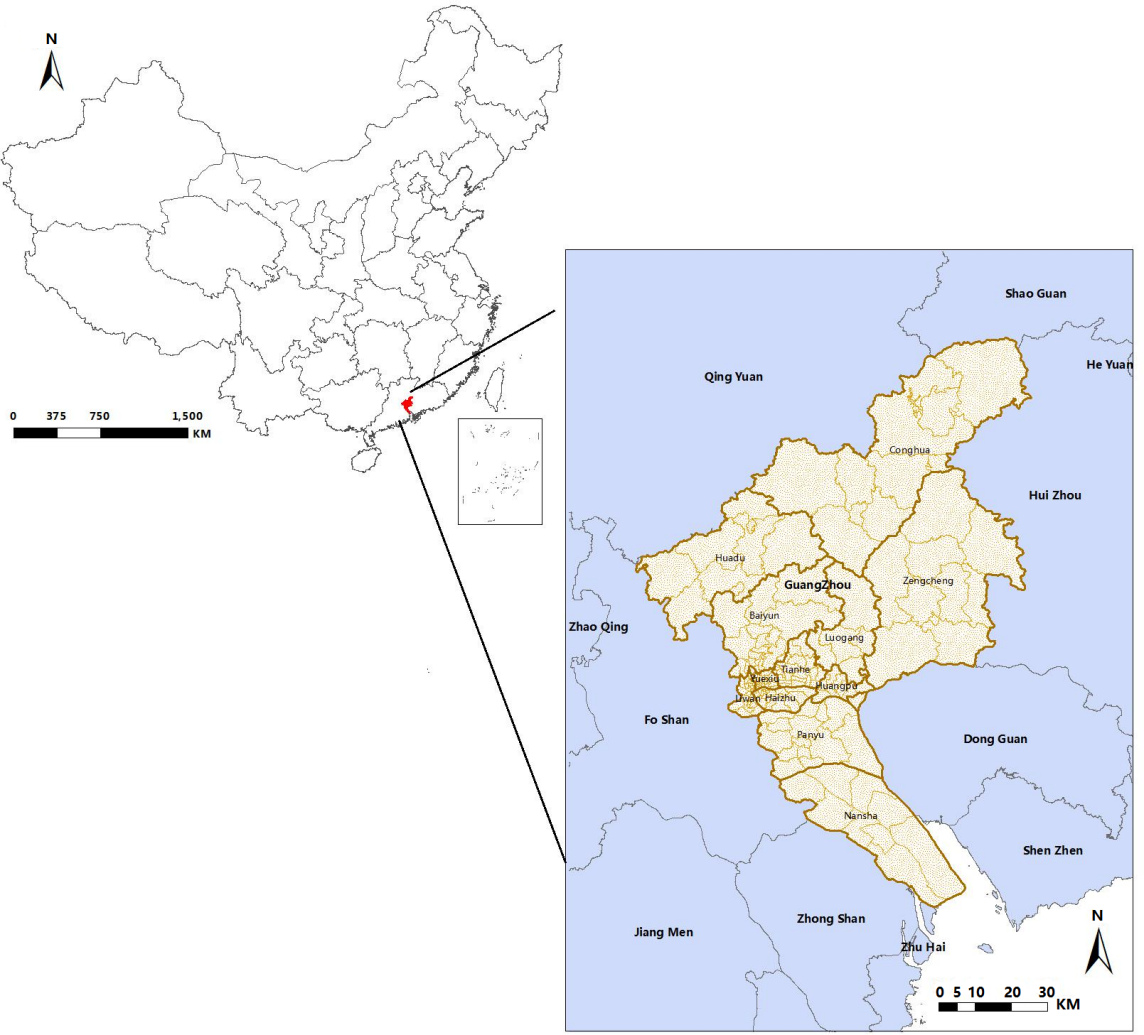
Location of Guangzhou city in Guangdong province, China. Fig 1 was generated by ArcGIS 10.0 (Environmental Systems Research Institute, RedLands, California, U.S.A).

Guangzhou, as the third-largest city in China and the world-famous trade port, located at the Pearl River Delta Region of Guangdong province and spanned from 112° 57' to 114° 03' E longitude and 22° 26' to 23° 56' N latitude [14] (Fig 1). The total area under the city's administration is 7,434.4 square kilometers and the permanent resident population is 12,700,800 (2010) [15]. Guangzhou city has 12 districts and 166 streets. The permanent resident population of each street ranged from 3397 to 391287 (2010) [16]. Monthly averages range from 13.6°C in January to 28.6°C in July, while the annual mean is 22.6°C [14], the relative humidity is approximately 68%, whereas annual rainfall in the metropolitan area is over 1,700 mm [14].

### Data collection and management

Daily data on indigenous DF cases were collected from China Notifiable Disease Surveillance System and Guangzhou Center for Disease Control and Prevention (CDC) for the years 2006 to 2014. There were 240 cases with unknown street-level address in 2014. These cases were excluded in this study. DF cases were diagnosed according to the national diagnostic criteria of DF, including the epidemiological exposure history, clinical manifestations and laboratory confirmation [10].

The street-level geographic vector polygon map of Guangzhou city was obtained from Guangzhou CDC and the latitude and longitude of the centroid of each street were calculated directly in the ArcGIS 10.0 software. The counts number of the indigenous DF cases were aggregated to counts at the street-level. Street-wise socio-demographic data was retrieved from the demographic bulletin of the 6^th^ National Population Census [17]. Data on the urban-rural structure of communities was collected from the National Bureau of Statistics of People's Republic of China [18]. The location of all cases were matched to the street-level vector map based on their home addresses.

### GIS visualization and spatiotemporal cluster analysis

The annual occurrence of street-wise first indigenous DF cases were mapped along with the date of onset.

A retrospective spatiotemporal scan test was implemented using SaTScan (Version 9.4.1) software. Firstly, the spatiotemporal cluster analysis of DF in Guangzhou from 2006 to 2014 was conducted annually. In brief, DF case, population and coordinates data were used as inputs in SaTScan.

Scanning window for the spatiotemporal scanning method is the spatial scan combining with temporal scan. The scan window is a cylinder. The base of the cylinder is circle which represents the spatial dimension, and the height of the cylinder represents the temporal dimension. The radius of the circle varied from zero to the maximum spatial cluster size of 50% of the population at risk which could avoid pre-selection bias. In this study, the heights of the cylinder were varied daily from zero to 1 year. The results with the statistical significance of *p*-value were reported by Monte Carlo simulation replication at 9999. The maximum log likelihood ratio (LLR) calculated in Poisson distribution is considered as the most likely cluster.

The secondary clusters are defined as the second maximum LLR estimated by poisson model [19].

In this study, a holistic purely spatial cluster analysis from 2006 to 2014 was implemented with the same upper limits in the spatial window. ArcGIS (Version 10.3.1) were used to convert the outputs of scan analysis into maps and visualize the spatial and temporal clusters.

### Socio-environment factors analysis of DF clustering

Univariate logistic regression and a stepwise logistic regression model were conducted to explore the relationship between the socio-environmental risk factors and the street with DF cases at high risk and low risk. Dichotomous dependent variable was set based on relative risks (RRs) of each street from the purely spatial cluster analysis result. The streets with RRs ≥1 were assigned “1” and those with RRs <1 were assigned “0”. The potential socio-environmental risk factors included at street-level were as following: percentage of people in each age-group; floating population; non-agriculture population; percentage of people with lower education (lower than undergraduate); percentage of different type communities (urban communities, urban-rural communities and rural communities) in all of the communities in each street. The floating population is defined as the people living in the street currently whose census registers were recorded in other street of the district in Guangdong province. There are two type of the census registers including agriculture and non-agriculture in China. The non-agriculture population was defined as the people whose census registers were recorded in the urban, not in the rural.

### Spatial variation analysis of DF transmission

The variations in the distribution of DF along the latitude and longitude of streets centroids were detected using Poisson model during the study period [20]. To explore the difference of DF distribution in the last three years and the first six years, we divided the study period into two periods: period 1 is from 2006 to 2011 and period 2 was from 2012 to 2014. The dependent variable in this modeling was the differences of DF annual mean incidence rates of the all the streets which occurred DF epidemic between the period 1 and period 2 in Guangzhou.

## Results

### Dengue epidemics and outbreaks

**Fig 2:**
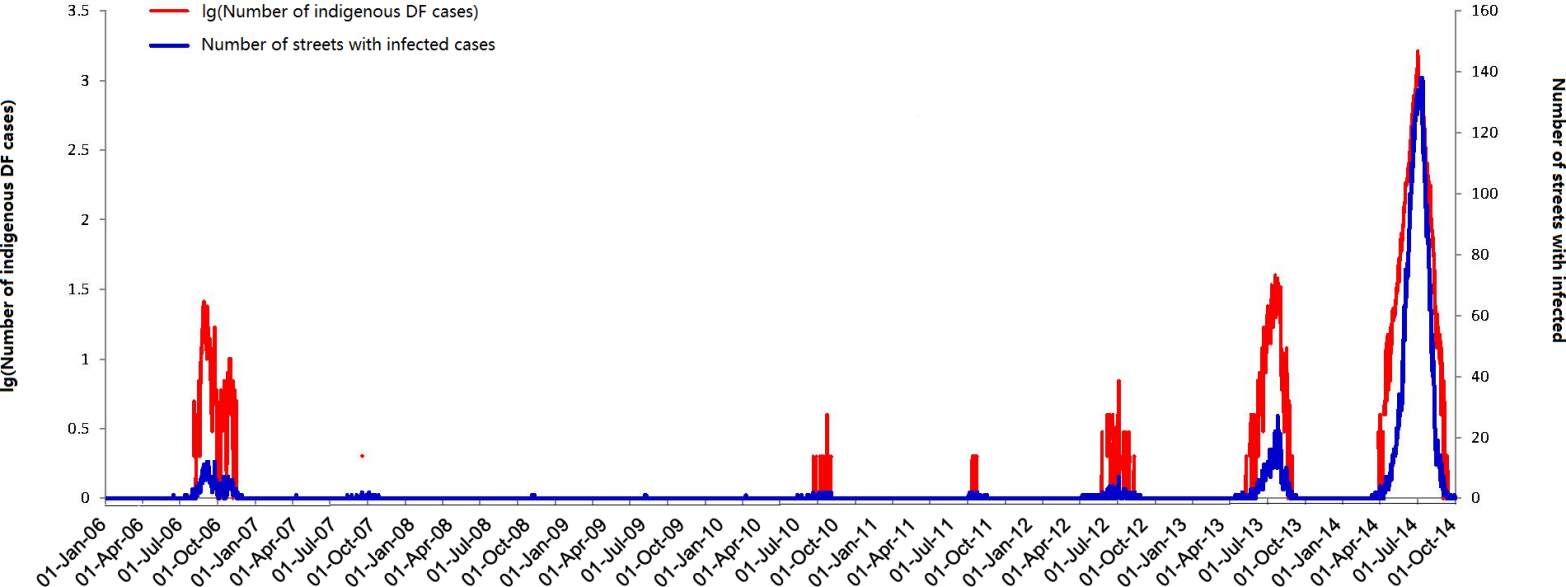
Daily number of streets with DF cases in Guangzhou city, China, between 1 January 2006 and 31 December 2014.

The epidemic pattern of daily indigenous DF cases fluctuated during 2006 to 2014 with three major outbreaks in 2006, 2013 and 2014 (Fig 2). The number of DF cases ranged from 0 to 1,627 cases daily (mean = 52.9, SD = 182.48). Interestingly, outbreaks showed an increasing trend after 2010. Fig 2 also displayed the daily variability of the number of streets with infected cases from 2006 to 2014. The peaks in DF cases generally coincided with streets of high DF cases.

**Fig 3:**
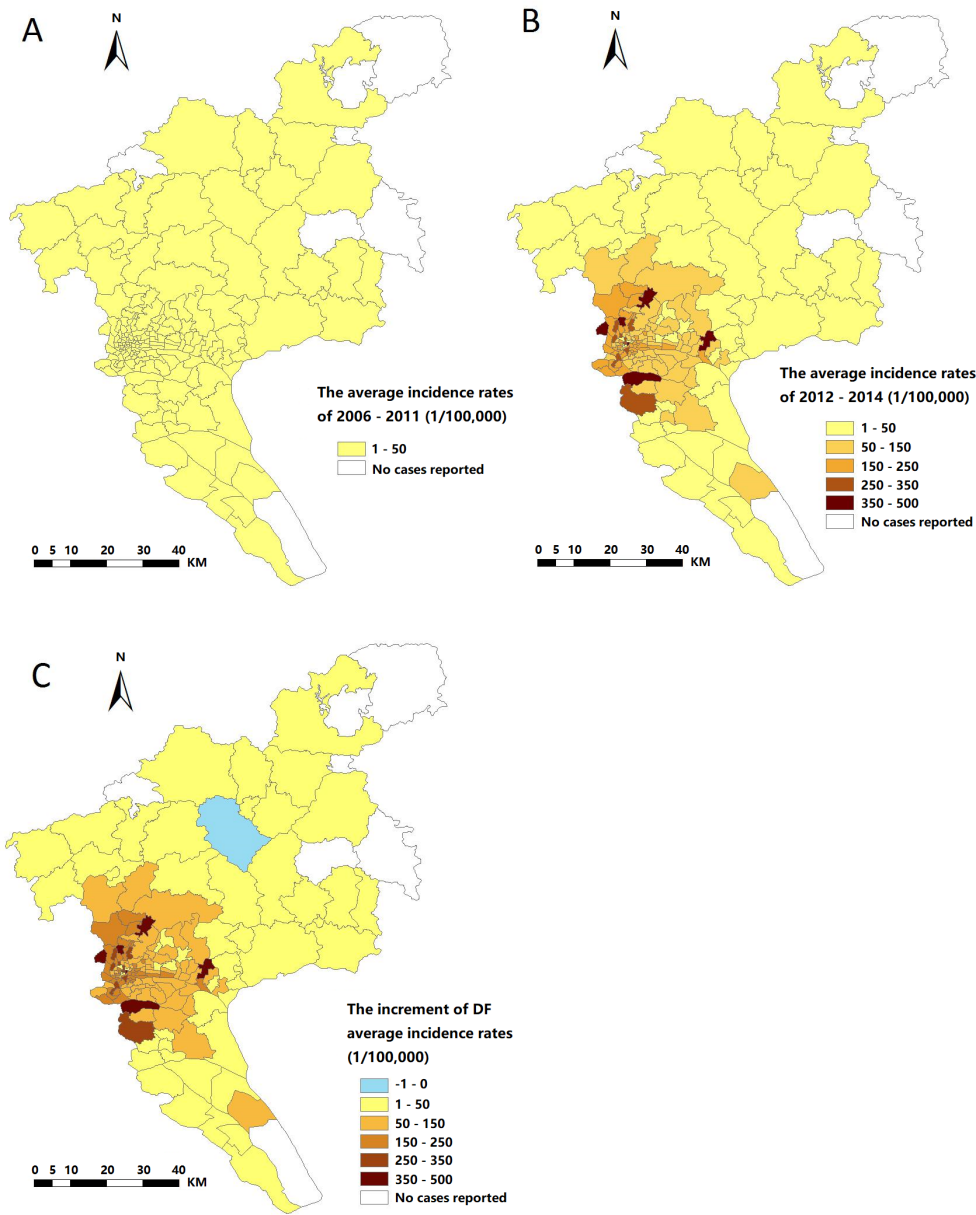
The average DF incidence rates and increment of average DF incidence rates. A, B, &C were generated by ArcGIS 10.0 (Environmental Systems Research Institute, RedLands, California, U.S.A.). The spatial distributions for annual mean DF incidence rates during 2006±2011; (B) The spatial distributions for annual mean DF incidence rates during 2012±2014; (C) The spatial distribution for increment of annual mean DF incidence rates from 2006±2011 to 2012±2014.

The spread of indigenous DF incidence rates in each high-risk street was displayed in Fig 3. All streets in Yuexiu, Liwan and Haizhu district, several streets in Baiyun, Panyu and Tianhe districts and streets in Huangpu, Luogang and Nansha district had relatively high DF spread. The streets with highest increase in DF were located in Baiyun, Panyu and Huangpu district. Baiyun districts included the streets with highest spread.

**Fig 4:**
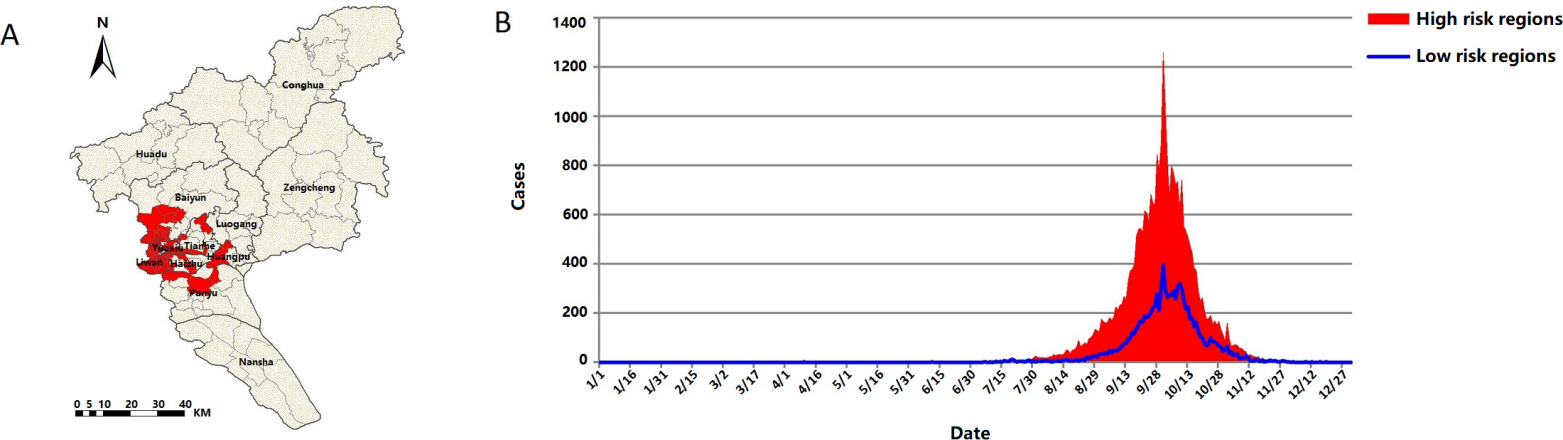
The spatial distributions of high and low DF risk streets and the counts of daily indigenous DF cases in high and low risk streets. Fig 4A was generated by ArcGIS 10.0 (Environmental Systems Research Institute, RedLands, California, U.S.A). (A) High risk streets with RRs>1 and low risk streets with RRs≤1. (B) The epidemic pattern of daily accumulative dengue cases in high-risk clustering streets and the low-risk clustering streets in Guangzhou city, 2006–2014.

Fig 4A showed the spatial distribution of high-risk areas or clusters of DF at street-wise. There were 75 high risk streets (RRs ≥ 1) in the southwest of Guangzhou city. These streets were located mostly in Yuexiu, Liwan and Haizhu district, the southern part of Baiyun, the northern part of Panyu, Tianhe and Huangpu district. Fig 4B depicts the sum of daily indigenous DF cases of the streets with RRs <1 and RRs ≥ 1 during the study period.

### Spatiotemporal cluster analysis and GIS visualization

**Fig 5:**
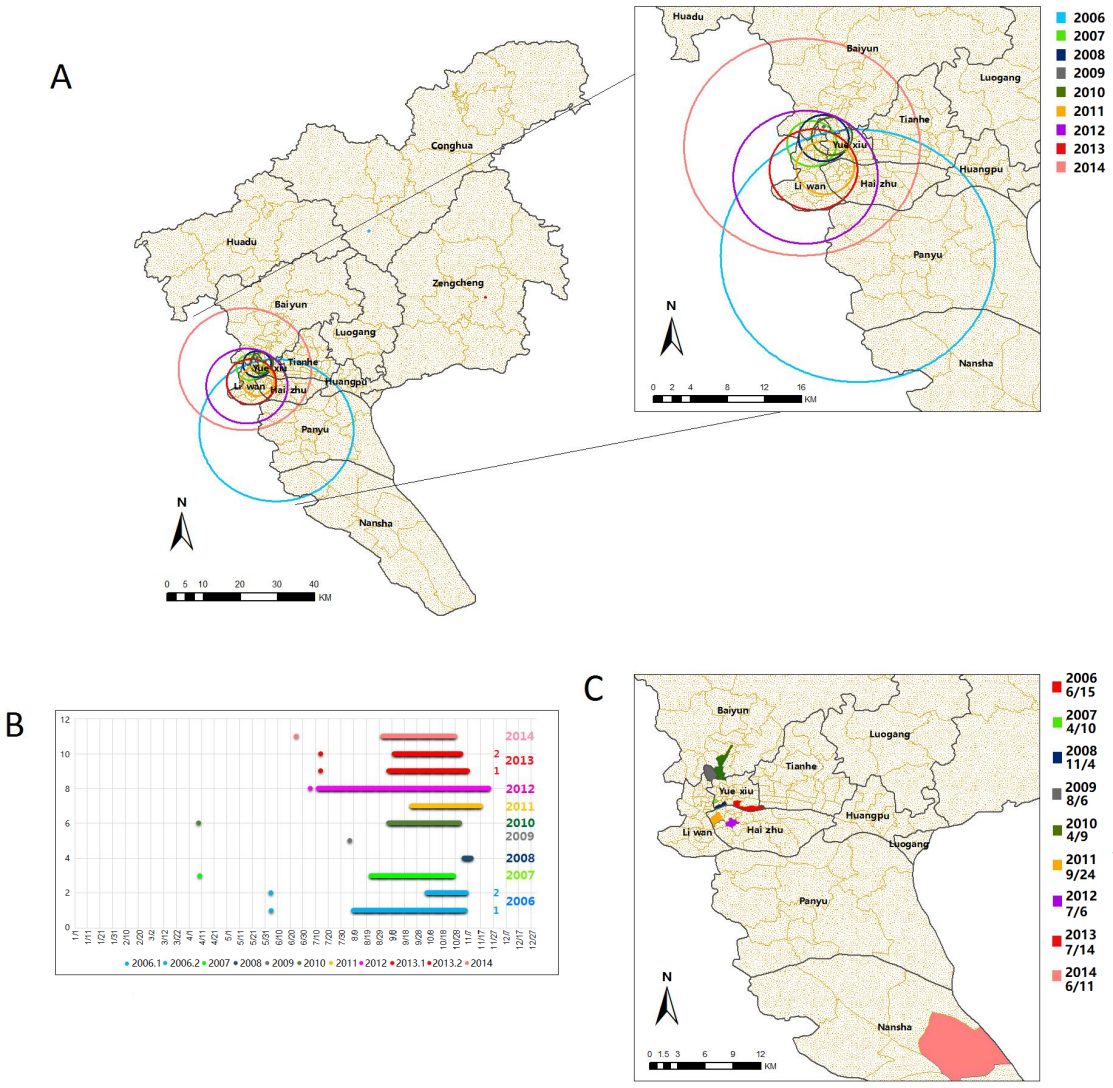
The spatial and temporal clusters of daily indigenous DF cases in Guangzhou city, 2006–2014. A&C were produced by ArcGIS 10.0 (Environmental Systems Research Institute, RedLands, California, U.S.A). (A) Spatial cluster circles of each year produced by SaTScan were displayed in different color. Both the most likely cluster and the secondary subcluster were displayed. Only the most likely clusters were displayed in the enlarged map. (B) Time clusters of each significant cluster of each year were plotted by different color and a dot in the earliest date denote the date of the onset of the first case of each year. In the year 2006 and 2013, “1” is the most likely cluster and “2” is the secondary likely cluster. (C) The streets where the first cases occur were plotted in different color and the date of onset were labeled below the legend.

Spatial and temporal clusters of indigenous DF cases were showed in Fig 5A and 5B, respectively. The most likely clusters (n = 9) were detected each year during 2006 to 2014 (*P*<0.01) and the secondary clusters (n = 2) were identified in 2006 and 2013 (*P*<0.01) (Table 1). The most likely clusters were concentrated in streets of Yuexiu, Liwan and Haizhu districts. In 2006, the most likely cluster included the southern Panyu district and part of the southern Nansha district whereas the secondary cluster included the northern Conghua district. In 2014, the most likely clusters included the farther northern Baiyun district with the secondary clusters in the northern Zengcheng district (Table 1).

**Table 1:** Cluster statistics from spatiotemporal cluster analysis from 2006 to 2014.

Year	Type of cluster	Number of streets in the cluster	Radius (km)	Duration	Population	Relative risk	Log likelihood ratio	P value
2006	M *	86	19.20	8/9 to 11/5	5,678,728	31.01	970.97	<0.01
	S *	1	0	10/6 to 11/6	94,369	102.43	173.13	<0.01
2007	M *	22	3.46	8/23 to 10/27	990,907	159.75	54.14	<0.01
	S	15	14.74	9/27 to 10/13	1,449,414	28.07	6.91	0.988
2008	M *	24	3.52	11/4 to 11/10	1,146,818	infinity	19.08	<0.01
2009	M *	1	0	8/6 to 8/6	90,036	154475.58	30.30	<0.01
2010	M *	17	2.85	9/6 to 11/1	825,057	168.55	135.25	<0.01
	S	75	53.25	9/20 to 10/26	6,096,390	5.95	12.02	0.228
2011	M *	33	4.06	9/24 to 11/17	1,685,967	489.98	107.37	<0.01
2012	M *	63	10.11	7/12 to 11/24	3,782,842	102.87	247.06	<0.01
2013	M *	45	6.15	9/6 to 11/7	2,176,701	82.38	2408.41	<0.01
	S *	1	0	9/10 to 11/2	191,777	9.63	35.36	<0.01
2014	M *	90	16.49	9/1 to 10/28	6,322,401	29.59	46332.28	<0.01

M, the most likely cluster; S, the secondary cluster

*, *P*<0.01.

The significant temporal clusters were found in autumn season, i.e., late August to early November during 2006 to 2014, except in 2008 and 2009. Fig 5C shows the streets with the occurrence of first indigenous DF cases each year. The first indigenous DF cases occurred within or close to the spatial cluster circles yearly, except in 2014, where it occurred in the distant Nansha district.

### The relationship between socio-environmental factors and DF epidemics

The results of univariate and step-wise logistic regression model analyses were presented in Table 2. In the univariate analysis, the age-groups, the percentage of non-agricultural population and the urban-rural population per street had significant association with DF risk: 0–14 years (OR = 0.84, 95% CI = 0.75 to 0.94), 15–64 years (OR = 0.94, 95% CI = 0.88 to 0.99), urban-rural communities (OR = 0.97, 95%CI = 0.95 to 0.98) and rural communities (OR = 0.95, 95% CI = 0.93 to 0.97) had negative association with DF risk whereas 65+ years (OR = 1.26, 95% CI = 1.15 to 1.39), nonagricultural population (OR = 1.05, 95%CI = 1.04 to 1.07) and urban communities (OR = 1.03, 95% CI = 1.02 to 1.05) had positive association with DF risk. After the stepwise variable selection, four variables were entered into the multivariate logistic regression model. The results demonstrated that DF was statistically significantly associated with population belonging to 65+ years (OR = 1.49, 95% CI = 1.13 to 2.03), floating population (OR = 1.09, 95% CI = 1.05 to 1.15), non-agricultural population (OR = 1.07, 95% CI = 1.03 to 1.11) and low-education population (OR = 1.08, 95% CI = 1.01 to 1.16).

**Table 2:** The associations between DF and social-environmental factors.

Independent variable (Percentage)	Univariate Logistic regression results		Stepwise Logistic regression results	
	OR	95% Confidence Interval	OR	95% Confidence Interval
Age group 0–14 **	0.84	0.75 to 0.94	-	-
Age group 15–65 *	0.94	0.88 to 0.998	-	-
Age group 65 + **	1.26	1.15 to 1.39	1.49	1.13 to 2.03
floating population (Street)	1.00	0.99 to 1.02	1.09	1.05 to 1.15
floating population (City)	1.00	0.98 to 1.01	-	-
Nonagricultural population **	1.05	1.04 to 1.07	1.07	1.03 to 1.11
Low-education population	1.00	0.97 to 1.03	1.08	1.01 to 1.16
Urban communities **	1.03	1.02 to 1.05	-	-
Rural-urban communities **	0.97	0.95 to 0.98	-	-
Rural communities **	0.95	0.93 to 0.97	-	-

*, *P*<0.05

**, *P*<0.01.

A statistically significant and negative association was obtained between the spread of DF incidence rates and longitudes (β = -5.08, *P* < 0.01) and latitudes of the streets (β = -1.99, *P* < 0.01) (Table 3). The results indicated that DF incidence rates increased with the areas geographically variation which may provide with the information of target streets for DF prevention and control in the future.

**Table 3:** The association between DF incidence rates and longitude and latitude geographically in each street during the study period.

Independent variable	Regression coefficients	Robust SE	95% Confidence Interval
Longitude **	-5.08	0.65	-6.35 to -3.81
Latitude **	-1.99	0.63	-3.24 to -0.75
Intercept **	615.03	76.3	465.49 to 764.57

**, *P*<0.01.

## Discussion

The results of this study suggested that DF incidence rates in the different districts appeared to be heterogeneous which was due to the changes in the transmission pattern of DF spatially and temporarily. A previous study has indicated that the prevention and control strategies towards DF will depend on high-risk and low-risk clusters [21]. Understanding and identifying the potential spatial and temporal clusters of DF transmission is the fundamental measure for surveillance and control [22]. A couple of studies have conducted cluster analysis of DF in Guangdong [23,24]. Previous research identified six risk factors for DF infection in Pearl River Delta [25] based on 2013 dengue surveillance data, which may improve our comprehension of the differences and socio-environmental factors on DF incidence rates. But in addition, few other studies have demonstrated that socio-demographic factors, such as population growth, levels of education, demographic structure and urbanization could influence the DF spread [26–30]. However, our research used a dynamic spatial and temporal analysis based on long term data (ie., January 2006 and December 2014) to detect the spatial clusters of DF and identify associated socio-environmental factors at a street level in Guangzhou. Moreover, Guangzhou was struck by an exceptionally severe outbreak in 2014, resulting in almost 40,000 laboratory-confirmed DF cases. This outbreak is the largest and most severe epidemic of dengue fever ever documented in China, with incidence rates exceeded the combined total of all previous years [31,32].

This study detected spatial clusters of DF high risk regions in Guangzhou city and suggested the geographic range of notified dengue cases has significantly expanded over recent years. Relative importance of risk factors may vary across space and time. This finding will provide useful information for developing dynamic early warning system for DF transmission. We have performed stepwise logistic regression model as this technique was applied in the vector-borne diseases research. Our results demonstrated that old aged population (65+ years), floating population, low-education people and non-agriculture people were the potential determinants for the spread of DF.

DF transmission has been reported in both rural and urban areas, and the dengue viruses have fully adapted to a human-*Aedes aegypti*-human transmission cycle, previous studies showed that the urbanization was linked to the DF incidence rates [33,34]. Guangzhou, as a large urban center of the tropics, where crowded human populations, especially nonagricultural population, live in intimate association with equally large mosquito populations. This setting provides the ideal home for maintenance of the viruses and the periodic generation of epidemic strains. In this longitudinal study, the result indicated nonagricultural population was positively related with DF risk, the central urban area and the old city area were the high-risk areas, where most aged (65+ years) Guangzhou residents lived. The streets with high nonagricultural population in Guangzhou normally have higher population density and poor housing conditions and less environmental management.

Previous studies have suggested that the accumulation of a susceptible population was essential to trigger DF epidemics [35]. In this study, a large number of floating population may be more susceptible for DF transmission. Residents, especially the aged, have the habit of planting flowers or hydrophyte in flowerpots or in household courtyards in Guangzhou. Several studies have identified the vegetation and breeding mosquitoes to DF that “vegetation can provide resting or feeding sites for mosquitoes or can serve as a proxy for the presence of breeding sites." Water storage, containers with an abundance of organic matter (e.g. those used for striking plant cuttings) or those amongst foliage or under trees (e.g. discarded plastic). As such progeny have been linked to a greater risk [36]. These containers with water provide a suitable breeding condition for mosquitoes. The water landscape and afforest landscape around the houses were also a perfect breeding habitat for mosquitoes. In addition, the movement of aged population may be limited to house surroundings and nearby areas, thus, increasing the chances of exposing themselves to mosquitoes. People with low-education generally have lack of knowledge and practices on the prevention measures of DF. These people usually work as laborers and spend most of their time outside, this in turn, may have given the possibility of being bitten by the mosquitoes. Another possible reason could be that these people live in rented apartments where the sanitary conditions are sub-optimal, thus this may have increased the chances of mosquitoes breeding and exposure.

The results from temporal cluster analysis indicated that the DF clusters occurred mainly in autumn, particularly, in late August to early November. Indigenous DF cases peaked seasonally despite limited intra-annual climatic variability and seasonal fluctuations. In addition, the availability of immature densities of *Aedes albopictus* (primary vector in Guangzhou) was consistent with the dengue seasonality [37,38] as the vector biology and viral replication are temperature and moisture dependent [39,40]. These results could be used in planning future prevention and control measures towards DF, particularly, during the high-risk season.

The consistent occurrence of first indigenous DF case within or close to the spatiotemporal clusters during the study period, except in 2014 requires further investigation. Over all, in the high risk streets, there were more indigenous DF cases than in the low risk streets: The cases in high risk streets occurred earlier and accelerated faster than those in the low risk streets as well. Without considering the number of cases, similar waves and crests were found in 2 sorts of streets. This could be due to the daily movements of working people from their living areas to working areas, i.e., the high-risk areas.

We observed an interesting result in the epidemic patterns of DF incidence rates during the study period. If the first case occurred in early summer, i.e., June or July, large outbreaks often occurred. For example, large epidemics in 2006, 2012, 2013 and 2014 were initiated with the occurrence of first case in June, July, July and June respectively. Although there were not many DF cases in 2012, the longest cluster period of DF was observed. On the contrary, if the first case occurred too early and too late, the large outbreaks often could not be triggered. In 2007 and 2010 epidemics, the first case occurred in April whereas in 2008, 2009 and 2011 epidemics, the first case occurred in November, August and September. If the first case occurs too early, the local department of health may plan to provide early warnings of DF outbreaks and implement prevention and control measures, whereas if it occurred too late, the reduced density of mosquito and the capacity of virus loading could help to decrease the risk of a large DF outbreak. Although imported cases was considered as an important trigger for the DF outbreak in Guangzhou, scientists could not confirm whether or not the dengue outbreaks in Guangzhou were initially triggered by the imported cases [39]. So other uncertainties of DF outbreak are still unknown and needs further studies.

In recent years, the impact of climate change on the transmission of mosquito-borne diseases has been studied in China [40]. Our results showed significant variation in the spatial distribution of DF in Guangzhou and that the geographic range of notified cases has expanded in this city (from south towards north and concentrate on the southwestern Guangzhou city) over the study period. Previous study reveal the movement tracks of the centre of mass for annual incidence rate of DF at municipality level in China, showing that the geographic expansion of dengue epidemics, such as gradually shifting from southern China (Guangdong, Guangxi, and Hainan) to northeastern China (Fujian and Zhejiang) and southwestern China (Yunnan) [41]. The associations between the spread of DF incidence rates and longitude and latitude were observed in this work, also demonstrated that DF has spread towards the southwestern Guangzhou city during the study period. Dengue is a complex disease and the spatiotemporal distribution involves socio-environmental factors, such as climate change, population movement, mosquito density and urbanization. Hence, future studies should include the impact of climatic and entomological factors on the transmission of DF in Guangzhou city.

To our knowledge, this is the first study to investigate the spatiotemporal clusters of DF and assess the socio-environmental factors in Guangzhou city using the spatial techniques at street-level. The study provides readily accessible information on DF spread and GIS maps on high-risk areas which can be used by the local Department of Health towards prevention and control of DF in Guangzhou. There are two limitations in this study: 1) Model included few variables on socio-environmental factors, as it was difficult to obtain all other street-level data. 2) As this study is an ecological study, measurement and information biases are possible. For example, the data on the socio-demographic factors were only obtained from the 6th Nation Population Census (collected in 2010) as the national demographic census in China was only conducted once 10 years. The socio-demographic data varied by time in Guangzhou and may have little impact on our results. However, we believe that the relative changes by different street level is unlikely to change dramatically in Guangzhou. We obtained the floating population in Guangzhou between January 1st 2006 and December 31st 2014 by accessing the registers at the online Guangzhou Statistics Bureau website (http://www.gzstats.gov.cn/). In addition, under-reporting is most likely possible as people with sub-clinical symptoms usually do not seek medical attention. The biases and drawbacks of stepwise multiple regression are well established within the statistical literature, including bias in parameter estimation, inconsistencies among model selection algorithms, etc. Whittingham et, al. discussed these issue and showed that stepwise regression allows models containing significant predictors to be obtained from each year's data [42]. In this study, we conducted stepwise logistic regression model as this technique was applied in the vector-borne diseases research, so as to select the main risk factors and develop predictive model. The spatial-temporal analysis presented in this paper differs from the one by explaining the observed distribution and perhaps ultimately permitting prediction.

In conclusion, this study has detected spatiotemporal clusters and variation of DF epidemics, and assessed socio-environmental risk factors for DF in Guangzhou city. These results could be implemented towards prevention and control measures of DF in high-risk areas in Guangzhou.
